# A Breakthrough in the Era of Calcium Silicate-Based Cements: A Critical Review

**DOI:** 10.7759/cureus.28562

**Published:** 2022-08-29

**Authors:** Payal S Chaudhari, Manoj G Chandak, Akshay A Jaiswal, Nikhil P Mankar, Priyanka Paul

**Affiliations:** 1 Conservative Dentistry and Endodontics, Sharad Pawar Dental College, Datta Meghe Institute of Medical Sciences, Wardha, IND; 2 Public Health Dentistry, Sharad Pawar Dental College, Datta Meghe Institute of Medical Sciences, Wardha, IND

**Keywords:** bioactivity, properties, clinical applications, dental materials, biocompatibility, endodontics

## Abstract

Calcium silicate-based cements (CSCs) or mineral trioxide aggregate (MTA) lookalike materials are blocks of cement or root canal sealers produced from calcium (Ca) and silicate. They have superior sealing ability, bioactivity, and marginal adaptability, making them appropriate for various dental treatment applications. Mineral trioxide aggregate is widely used in numerous endodontic repair techniques. The capacity of this cement to promote tissue regeneration and stimulate mineralization accounts for its widespread usage in pulp capping, apexification, apical surgeries, and revascularization. Several studies have been conducted to investigate changes in the components of MTA-based types of cement directed to improve their presentation clinically. To improve flowability, new Ca silicate-based formulations have been introduced commercially. In these new formulations, essential features such as adequate radiopacity and setting time, color stability, alkaline pH, and calcium ion release and biocompatibility must be considered. Owing to an increased range of indications of CSCs, including some for restorative dentistry, and with the emergence of novel silicate calcium-based materials with considerable changes in their compositions, it is necessary to examine the available scientific literature that evaluates their usage in these applications. Therefore, this review paper aims to assess the existing knowledge of CSCs, emphasizing their potential uses in restorative and endodontic dentistry. This report strives to update doctors' understanding of CSCs, allowing for a better therapeutic approach.

## Introduction and background

Reparative techniques are vital in endodontics, and conservative measures help preserve the vitality of teeth and ensuring they are in good health [1,2]. Mineral trioxide aggregate (MTA) is a biocompatible compound that has found widespread usage in clinical endodontic therapy because of its low cytotoxicity and high biocompatibility and ability to stimulate new dentin development. It has been the material of paramount importance since its introduction in the 1900s [3]. Uses of MTA include conservative management of root fractures, perforation repair [4], pulp capping agent [5], apexification [6], retrograde filling material in apical microsurgeries [7], and revascularization measures as a coronal barrier [2]. The above procedures involve close contact with the body fluids and vital tissues, favoring physical alterations and chemical/biological communications with the material [8].

Various properties of MTA, such as physical, chemical, and biological, have been explored for extended periods, leading to discoveries of its efficient substitutes. However, improvisations are still needed to arrive at an ideal composition of the constituents of the cement. The development of a model, unflawed restorative material is still long due. To achieve this it should possess the following characteristic properties: sealing ability, dimensional [9] and color stability [10], radio-opacity [11], insolubility when in contact with body fluids, and ability to flow with easy insertion. It should also possess biological and chemical properties such as alkaline pH, calcium (Ca) ion release, bioactivity, cell attachment, and biocompatibility [12]. Mineral trioxide aggregate owns most of the mentioned ideal properties but lacks a few, primarily color and consistency which require the most improvisations [13]. Therefore, materials with newer innovations have been launched commercially to overcome these shortcomings. This review intends to highlight the properties of MTA with their limitations and to arrive at the developments in innovative Ca silicate-based cements (CSCs).

## Review

Clinical properties and characteristics

The main emphasis should be on the clinical facet of these CSCs, as the site of placement directly influences and determines material properties [14]. Mineral trioxide aggregate is a dynamic, active material as its application and placement result in constant contact of the cement with tissues and fluids. It persists for years after its insertion [15]. Its mechanism comprises Ca hydroxide leaching out of the hydrated MTA, thereby highlighting the bioactivity of MTA, which relates to the calcium ion (Ca2+) release. Placement of MTA is usually required at the site where there is a presence of blood that contaminates it, affecting the structure of the set material and reducing the Ca2+ release [6,16]. The principal limitations of MTA include a delayed setting time, lack of good handling features, and the disadvantage of discoloration.

Also, the contact of MTA with blood can alter the color of the material and interferes with radiopacity over time [17]. Moisture drastically affects the time of setting and the material solubility. Excessive water results in increased solubility and setting time of MTA. During the setting process of MTA, it chemically interacts with tissues making the environment alkaline by releasing Ca2+ ions, which are linked to the development of portlandite (calcium hydroxide) by tricalcium silicate (C3S) and dicalcium silicate (C2S) [18].

In vitro studies done with MTA Angelus and ProRoot MTA revealed Ca2+ ion discharge and alkalization of the environment when the material was submerged in water. The release of Ca2+ ions was detected by von Kossa staining of subcutaneous tissues of rats [19]. These properties lead to mineralization on the surface of the set MTA in pulpotomy procedures. This is proven by studies where hard tissue was formed apically in a dog's teeth which were seen along with the sealing ability in cases of furcation perforation [20].

In an in vivo study by Han et al., the odontogenic potential of osteostatin (OST) and the combined effect with bioceramic materials on human dental pulp stem cells (hDPCs) were investigated, and it was discovered that the combination of MTA and OST had a synergistic odontogenic differentiation of hDPCs when compared to MTA alone [21]. Micro-CT research demonstrated that OST with ProRoot MTA groups formed more mineralized dentin bridges [22].

Color stability

During dental operations, the most significant property of observation is color. Tooth discoloration damages the tooth's aesthetic appearance. The complex response between filling materials and coronal dentine of the pulp chamber, which modifies the crown’s appearance, is a significant cause of tooth discoloration.

Initially, when developed, MTA had a grey color owing to the presence of tetra Ca aluminoferrite, making it unsuitable for its application on anterior teeth. Therefore, this led to the establishment of white MTA which is devoid of iron to prevent the discoloration of the tooth. On the contrary, many studies have proven the alteration in color even with white MTA [23]. The composition of white MTA includes C2S and C3S silicate with 20% of bismuth oxide. According to reports, the amount of bismuth oxide added to MTA to increase its radio-opacity was only 8.4% in the set material compared to 21.6% in the unset material [8]. When reduction of bismuth oxide occurs along with its contact with the tooth structure, it alters the color of the cement and the color of the adjacent tooth structure. The cause of color change has been identified and attributed to the loss of stability of the bismuth oxide molecules, which occurs as they come in close contact with a potent oxidizing agent [24]. Hence, it is suggested that if the radiopacifier agent is changed, it can help prevent the discoloration of the tooth. Two materials have been lab tested to replace bismuth oxide, namely zirconium oxide and Ca tungstate. However, large amounts are necessary to match the radiopacity of bismuth oxide. Adding such large amounts can negatively impact the chemical and physical properties of the dental material [25]. Newer CSCs such as Biodentine and Bioceramic (BC) sealer, and MTA high plasticity (HP) can alter the radiopacifier agent into Ca tungstate or zirconium oxide. These constituents caused no alteration in color [26]. The second substitute is the addition of 5% zinc oxide (ZnO) to MTA as this ZnO converts bismuth oxide into bismite, a product that helps prevent the change of color [10].

Consistency

There is a difference in opinion regarding the consistency of MTA. The ratio of powder to water is essential as increasing the quantity of water reduces radiopacity. The particle size is vital here as the newer advances in silicate types of cement have been developed using nanoparticles of Ca silicate (CS). The BC sealer and biosealer containing nanoparticles of CS with the addition of a polymer help in easy handling and give an ideal material consistency. Adding propylene glycol to MTA caused no interference in its biological properties. Propylene glycol was tested using different ratios for chemical and physical properties in which 20% propylene glycol was mixed with 80% distilled H2O, which led to efficient handling of MTA, pH, enhanced Ca release, and flowability. However, it caused slight alterations in setting time [27]. Few studies have proven that propylene glycol caused improved adhesion of MTA.

Newer preparations

The advances which lead to enhanced flow ability comprise MTA HP, MTA Flow, Biodentine (Septodont, Saint-Maur-des-Fossés, France), and ones having ceramic complexes incorporated with Biodentine, EndoSequence (Brasseler, Savannah, GA, USA), and BioAggregate (Verio Dental Co. Ltd., Vancouver, Canada).

In 2009, Biodentine, a Ca silicate-based product, was introduced. Zirconium oxide is used instead of bismuth oxide in Biodentine. Zirconium oxide is a bioinert substance with good mechanical qualities & corrosion resistance. Dettwiler et al. 2016 observed this closely in an experiment [28]. Biodentine had a minor discoloration, higher solubility than MTA, and a significantly faster setting time. In as little as 12 minutes, Biodentine can begin to block blood components, becoming denser and packed as it sets. As a result, erythrocyte penetration is reduced, resulting in less tooth discoloration during the pulpotomy operation. Because it comprises more powder with a water-reducing agent and less porosity, the Biodentine material significantly impacts various factors such as absorption, strength, and density [29]. Biodentine and zinc oxide-eugenol cement (IRM) had the lowest level or degree of porosity and the least amount of tooth discoloration, according to Camilleri et al. in 2013 [14].

Endosequence root repair material (ERRM), is available as a premixed putty with uniform consistency and easier handling and application. According to the manufacturer, the setting begins with the presence of moisture in the dentinal tubules. When pulp cells were exposed to ERRM or ProRoot MTA, survival and proliferation were identical, suggesting that it could be a good choice for pulp capping treatments [30].

BioAggregate (BA) contains monobasic Ca phosphate, amorphous silicon dioxide, and tantalum pentoxide for radiopacity. And due to its Ca phosphate content, it is classified as a biphasic material (one that contains two cementitious ingredients) [31]. It is more acid resistant than MTA, has a longer-lasting strengthening effect on weaker teeth, and has a lower risk of discoloration [31]. In the treatment of immature teeth, it has demonstrated similar results as MTA.

Biological properties of CS-based types of cement and newer advances

The main composition of MTA is CS. Bioactivity is one characteristic feature of Ca silicate-based types of cement [27]. Newer CS-based restorative types of cement have been launched to substitute bismuth oxide like Biodentine, Neo MTA Plus (Avalon Biomed Inc. Bradenton, FL, USA), and MTA Repair HP (Angelus, Londrina, PR, Brazil). Others include MTA Fillapex (Angelus, Londrina, PR, Brazil), Neo MTA Plus, iRoot SP (Innovative BioCreamix Inc, Vancouver, BC, Canada), and TotalFill BC (Davis Schottlander & Davis Ltd. Letchworth, Herts, UK) sealer.

The MTA Fillapex cement comes in a paste-paste form which comprises salicylate and natural resin, infused silica nanoparticles, MTA, and Ca tungstate which acts as radiopacifier. There is a newly introduced C2S silicate-based system with a powder-gel formulation named Neo MTA, a remarkable restorative and endodontic cement that can be used with various proportions of powder gel ratios. The composition of iRoot SP is zirconium oxide, CS, Ca phosphate, Ca hydroxide, and thickening agents, which are commercially accessible and is used as a root canal filling material. On the other hand, EndoSequence BC sealer and TotalFill BC sealer comprises zirconium oxide, CS, monobasic Ca phosphate, Ca hydroxide, and thickening agents. This latter cement is advantageous as it sets in the presence of dentin moisture and hence was used as canal filling material.

A study on iRoot SP endodontic cement advocated the absence of cytotoxicity to fibroblasts when tested in rats [32]. Alternative research by Zoufan et al., checked the cell compatibility of iRoot SP cement at two stages: after the cement was freshly mixed, and after the cement had been set [33]. It was found that this cement had a greater induction capacity of osteoblastic differentiation and decreased inflammatory response with the periodontal ligament cells compared to Sealapex [34].

The MTA and iRoot SP types of cement have been proven to induce differentiation in osteoblastic cells in the tooth germ. The iRoot SP significantly showed its antibacterial activity against *Enterococcus faecalis *[35]. Zhu et al. found evidence of the ability of BioAggregate cement to promote cell adhesion to each other, migration, and fixation of human dental pulp cells, thus proving its cytocompatibility [36].

Bioceramic endodontic cement-like Endosequence BC sealer has displayed promoting superior cell viability than AH Plus sealer and also offered an increased level of biocompatibility when compared with newly handled AH Plus and MTA Fillapex, when freshly mixed and after the setting. Bioceramic sealer has shown satisfactory adhesion to fibroblasts [37]. Upon contact with the biological solution, discharge of Ca and development of the Ca phosphate phase was seen. Antibacterial activity against biofilm formed on dentin was greater when Endosequence BC sealer was used along with 5% sodium hypochlorite than the irrigation solution alone [38]. In a study using confocal laser microscopy, Wang et al. concluded that in 30 days, a BioCeramic sealer could eliminate 45% of *E. faecalis* from the dentinal tubules, indicating the antibacterial action of the Bioceramic sealer lasted even after the setting of the material [39]. Total fill BioCeramic sealer is identical to Endosequence BC sealer. The only difference is that the former promotes extensively higher proliferation of cells compared to AH Plus and MTA Fillapex. The structure of cells embedded on Total Fill BioCeramic Sealer and AH Plus showed similar physiognomies, along with the assembly of the extracellular matrix. In contrast, limited fixation of cells was seen on discs of MTA Fillapex, with decreased number of cells on the material surface [40].

The MTA Angelus, MTA HP, and Neo MTA P presented viability of cells and a higher degree of cellular proliferation along with adhesion. Using HDPCs, greater viability was seen with MTA plus compared to MTA Fillapex and Fillcanal; increased phosphates activity was observed with MTA Plus [41,42]. No cytotoxic effect was seen with Neo MTA Plus, MTA Angelus, and experimental C3S silicate-based cement with tantalum oxide (TSC/Ta205). According to the alizarin red assay, the three materials were proven to induce the formation of mineralized nodules; on the other hand, NEO produced a considerable quantity of mineralized nodules compared to MTA and TSC [43]. Following subcutaneous implantation in rats, histological analysis established that MTA HP showed similar biomineralization and biocompatibility potentials to MTA Angelus [43]. The MTA Angelus and MTA Plus showed no presence of cytotoxicity and induced mineralized nodule formation. When PCR was used, the authors concluded that when HDCPs were exposed to extract the two types of cement, it increased the expression of osteogenic markers of the cell [44].

According to Petrovic et al., materials based on CS and hydroxyapatite (HA-CS) showed a superior grade of biocompatibility compared to MTA Angelus [45]. Also, improved outcomes were seen for CS and HA-CS when subcutaneous implants were placed in rats. In the assessment of the biocompatibility of three Ca silicate-based types of cement, which include Bioroot BC sealer (BR), Endoseal MTA (ES) & Nanoceramic sealer (NCS), along with human periodontal ligaments stem cells (hPDLSCs), BR and NCS showed superior cytocompatibility as compared to ES [46]. The BC sealer was proficient in hindering the release of immunoreactive calcitonin gene-related peptide (iCGRP) from trigeminal ganglion neurons and excellent biocompatibility, thereby reducing the symptomatology level after extravasation of the cement in ongoing treatment [47].

In a study by Almedia et al., a comparison of physiochemical and biological properties of already mixed Ca silicate-based endo sealers with routinely used root canal (RC) filling materials by thoroughly revising lab investigations [48]. Calcium silicate-based endodontic sealers follow the ISO 6876:2012 standard for most physicochemical properties, except solubility. The target sealers depicted commendatory biological traits in comparison to conventional sealers. Despite failing to test the target premixed Ca silicate-based sealers in long-term experimental clinical trials, they presented with good physicochemical and biological traits in vitro.

## Conclusions

Numerous formulations with added benefits have been introduced to surpass the shortcomings of MTA, and are commercially available. The newer advances including MTA HP, MTA Flow, Biodentine, and those having ceramic complexes incorporated in them, such as Biodentine and Endosequence, could serve as gifted substitutes to MTA. However, additional research assessing their clinical outcomes is essential. The revised formulations of CSC have identical elemental composition and biological proper; the only difference between them is in either setting reaction time and physical properties. However, these traits could be of lesser prime value when the use of these materials is confined to non-stress-bearing regions and multiple visit appointments. The deciding factor for the usage of specific cement for endodontic repair will rely on research quality investigating the clinical outcomes, site of application, strength, nature of overlying restoration, preliminary strength, and choice of the clinician.
